# Machine Learning Algorithms, Applied to Intact Islets of Langerhans, Demonstrate Significantly Enhanced Insulin Staining at the Capillary Interface of Human Pancreatic β Cells

**DOI:** 10.3390/metabo11060363

**Published:** 2021-06-07

**Authors:** Louise Cottle, Ian Gilroy, Kylie Deng, Thomas Loudovaris, Helen E. Thomas, Anthony J. Gill, Jaswinder S. Samra, Melkam A. Kebede, Jinman Kim, Peter Thorn

**Affiliations:** 1 Charles Perkins Centre, School of Medical Sciences, University of Sydney, Camperdown 2006, Australia; louise.cottle@sydney.edu.au (L.C.); kden4987@uni.sydney.edu.au (K.D.); melkam.kebede@sydney.edu.au (M.A.K.); 2 School of Computer Science, University of Sydney, Camperdown 2006, Australia; ian.gilroy@sydney.edu.au (I.G.); jinman.kim@sydney.edu.au (J.K.); 3 St Vincent’s Institute, Fitzroy 3065, Australia; tloudovaris@svi.edu.au (T.L.); hthomas@svi.edu.au (H.E.T.); 4 Department of Medicine, St Vincent’s Hospital, University of Melbourne, Fitzroy 3065, Australia; 5 Northern Clinical School, University of Sydney, St Leonards 2065, Australia; Anthony.Gill@health.nsw.gov.au (A.J.G.); jas.samra@bigpond.com (J.S.S.); 6 Department of Anatomical Pathology, Royal North Shore Hospital, St Leonards 2065, Australia; 7 Cancer Diagnosis and Pathology Research Group, Kolling Institute of Medical Research, St Leonards 2065, Australia; 8 Upper Gastrointestinal Surgical Unit, Royal North Shore Hospital, St Leonards 2065, Australia

**Keywords:** insulin, beta cell, human, islet, polarisation, machine learning, deep learning, cell segmentation, automation

## Abstract

Pancreatic β cells secrete the hormone insulin into the bloodstream and are critical in the control of blood glucose concentrations. β cells are clustered in the micro-organs of the islets of Langerhans, which have a rich capillary network. Recent work has highlighted the intimate spatial connections between β cells and these capillaries, which lead to the targeting of insulin secretion to the region where the β cells contact the capillary basement membrane. In addition, β cells orientate with respect to the capillary contact point and many proteins are differentially distributed at the capillary interface compared with the rest of the cell. Here, we set out to develop an automated image analysis approach to identify individual β cells within intact islets and to determine if the distribution of insulin across the cells was polarised. Our results show that a U-Net machine learning algorithm correctly identified β cells and their orientation with respect to the capillaries. Using this information, we then quantified insulin distribution across the β cells to show enrichment at the capillary interface. We conclude that machine learning is a useful analytical tool to interrogate large image datasets and analyse sub-cellular organisation.

## 1. Introduction

Defective insulin secretion from islet β cells is a characteristic feature of diabetes mellitus [1]. To better understand molecular mechanisms that regulate insulin secretion, we need to be able to image and study β cells and their subcellular structures [2], particularly their organisation within the native environment of the islets of Langerhans.

With the advancing capabilities of modern microscopy systems, the detailed visualisation of cells and their subcellular components is possible [2,3]. However, this has also led to the rapid generation of complex and ever-expanding image datasets [4,5]. The bottleneck facing researchers now is the extraction and quantification of valuable biological insights from these large image datasets [4,6]. Thus, the need for automated image analysis methods becomes increasingly important. Computational image processing methods have traditionally relied on static and predefined rules [3]. However, a major shortcoming of this approach emerges when these static algorithms are applied to different datasets or datasets of high variability, often requiring labour-intensive reprogramming and/or the manual adjustment of predefined parameters [3]. In contrast, machine learning seeks to detect patterns from training data, and then apply those patterns to new datasets [3,7]. With sufficient training data, encompassing wide variations in morphology, the same algorithm can be reused, even for different experimental setups, without the need for code tweaking [6,8]. This approach not only reduces human workload [9], but also offers significant advantages over conventional image processing methods in its ability to ensure objective, reproducible and timely analysis [3,6,10].

The last two decades have seen an expansion of machine learning applications in biological studies [4,5,11,12]. In particular, deep learning, a subtype of machine learning, has gained significant popularity in automated applications including image classification [5], tissue [13,14] and cell image segmentation [6,7], and nuclei identification and quantification [8]. A deep learning approach to image processing works by using neural network structures to extract features of a given image dataset in “layers” or levels of hierarchy [15]. Successive layers of representations are generated such that the higher levels of hierarchy are composed using the output of lower-level features [16]. Deep learning methods have demonstrated success at the cellular level in segmentation applications of a range of cell types, including bacteria and mammalian cells from phase contrast images [17], HeLa cells from DIC microscopy images [18], neuronal membranes in electron microscopy images [19], yeast cells [6], and circulating tumour cells [20]. At the subcellular level, deep learning algorithms have also precisely segmented the nuclei and cytoplasm in fibroblasts, HeLa, HepG2 cells [2,21,22].

Applied to the study of islets of Langerhans, automated analyses have been used for the segmentation of islets and pancreatic exocrine tissue [23], as well as the quantification of individual islets and islet cell density [24]. At the cellular level, however, only a few studies have applied machine learning methods to the study of islets. Human islets are composed of five endocrine cell types, insulin-secreting β cells (~65%), glucagon-secreting α-cells (~30%), somatostatin-secreting δ-cells (~5%), pancreatic polypeptide-secreting γ-cells and ghrelin-secreting ε-cells (<1%) [25,26]. The challenge of using machine learning for islet cell segmentation lies in the complex variation in structure and shape of these islet cells [27]. Not only is it difficult to distinguish between the different cell types, but cells are also often of irregular shape and closely packed together [27,28], leading to challenges in border detection between cells and the generation of labelled images for training models.

In the last decade, a small number of studies have highlighted the potential of automated image analysis methods for the segmentation of these islet cells. In 2012, an analytical software program, *Pancreas++*, was developed for the classification and positional quantification of α and β cells within islets in fluorescence microscopy images [29]. In another study, using the immunofluorescence staining of TMEM27 and BACE2 in islets, an automated image analysis pipeline was generated to determine β cell number, area and density per islet [23]. However, while these studies employ automated image analysis approaches, they largely focus on cellular arrangements within an islet, rather than individual β cells and their subcellular structures and protein distributions. Alternative computational approaches to study islets using mathematical modelling have generated three-dimensional reconstructions of pancreatic islets; however, these are not without limitations. For example, many models have not been able to accurately capture the heterogeneity of cell sizes and shapes within an islet [30,31]. In other models, the presence of various islet structures including vasculature have not been considered [32].

There are many aspects of the biology of β cells that could be advanced by machine learning approaches. For example, accumulating evidence indicates the presence of the structural and functional polarisation of β cells [33,34,35,36], reminiscent of cell polarity in epithelial cells [37]. Key regulators of cell polarity such as liver kinase B1 (LKB1) have been identified in rodent β cells [37], as well as cell polarity determinants including discs large (Dlg), partitioning defective 3 homologue (Par3) and scribble, showing consistent orientation with respect to islet vasculature in both human and rodent β cells [35,36]. Previous studies have also indicated β cell regional specialisations, such as the selective localisation of the GLUT2 transporter on the lateral membrane domain between adjacent β cells [38], as well as the targeting of insulin granule fusion at the vascular interface of the β cells [39]. The presynaptic scaffold proteins liprin, ELKS, Rab3-interacting protein (RIM2) and piccolo show enriched expression at the β cell–vasculature interface [36,39], suggesting that insulin secretion may be regulated by mechanisms similar to a neuronal synapse [39]. Furthermore, it has also been suggested that insulin content is asymmetrically distributed in the β cell, with an enrichment at the β cell–vasculature interface [36,40]. However, little is currently known about the mechanism linking β cell structural polarity and cell function.

Here, we use a deep learning approach to segment β cells, and subsequently investigate the subcellular organization of β cells within islets by analysing the distribution of insulin with respect to cell contacts with islet vasculature. We assessed two commonly used deep learning models for image segmentation applications, namely, the U-Net fully convolutional networks (FCN) and residual neural networks (RNN), for the automated segmentation of β cells from microscopy images of human pancreatic islet slices. We next applied the U-Net model to create β cell mask images, used to predict the location of β cells within islets. Analysis of insulin distribution in over 2000 β cell instances using computational techniques demonstrated an enrichment at the capillary interface of β cells.

## 2. Results

Human pancreas samples sourced from either partial pancreatectomy patients or cadaveric donors were processed using the pancreatic slice technique [41]. In this process, 150 µm sections were stained and imaged using 3D fluorescent microscopy. Deep learning approaches were undertaken on the resultant images, first to predict β cell locations and boundaries and then to assess subcellular fluorescent staining.

### 2.1. Manual Analysis Reveals Increased Insulin Staining at the Capillary Interface of β Cells

In situ analysis of β cells in islets within pancreatic slices provides evidence that β cells are polarised, and that both mouse and human β cells maintain a consistent orientation with respect to the vasculature [35,36]. The islet vasculature is composed of cells and secreted basement membrane, which is a complex mixture of proteins including laminin [42], and in this work we have used laminin-β-1 as a marker for the islet vasculature/capillaries.

**Figure 1:**
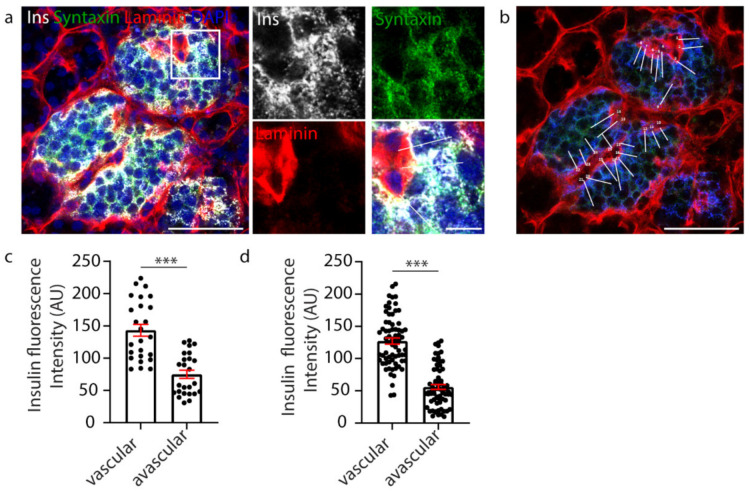
Manual analysis of β cell insulin intensity. (**a**) Representative image of a whole human islet with inset. Ins-grey, Syntaxin 1A-green, Laminin-red, DAPI-blue. (**b**) Whole islet image with line-scans (white) over β cells contacting the vasculature. Ins-blue, Syntaxin 1A-green, Laminin-red. (**c**) Graph of insulin intensity analysis for the islet in a-b, cells *n* = 25 *** *p* < 0.0001. (**d**) Graph of insulin intensity analysis for all β cells analysed. Data are representative of *n* = 3 donors (1–2 islets per donor), cells *n* = 83 *** *p* < 0.0001. Scale bar 50 µm on whole islet images and 10 µm on insets.

β cell orientation has important functional consequences, such as the precise targeting of insulin secretion to the vasculature [35,36]. A recent study, in mice, suggested the presence of a population of β cells with an asymmetric distribution of insulin content, showing an enrichment of insulin in the regions adjoining the islet capillaries and an avascular location for insulin mRNA [40]. Therefore, we investigated whether vasculature contact influenced insulin distribution within human β cells. In this study, human islets were immunostained to visualise the β cells (insulin), their cell boundaries (syntaxin 1A) and the surrounding vasculature (laminin) (Figure 1a). To assess insulin distribution, manual analysis involved assessing one z-plane of the islet and drawing a perpendicular line across each β cell from the vascular face (laminin) to the avascular face (opposite the vasculature), and the fluorescence intensity was measured at each face using a line-scan (white lines, Figure 1b). The results showed that insulin distribution across each β cell was asymmetric and enriched towards the vasculature (Figure 1c, *n* = 25). This relationship was consistently observed in all islets analysed (Figure 1d, *n* = 3 donors, 1–2 islets per donor, *n* = 83 cells) [36]. This analysis, whilst informative, was performed manually, and so was relatively labour-intensive and sampled only a subset of β cells in the islet.

### 2.2. U-Net-Based Deep Learning Was the Most Efficient for β Cell Segmentation

We set out to develop a new automated approach to provide a more objective, time-efficient analysis that would allow the inclusion of the majority of β cells within the islet volumes imaged. Previous studies have used automated approaches to assess islet cell density and islet cell proportions (α and β cells) with islet 3D reconstructions [24,29,32,43]. Here, we aimed to create an automated model capable of identifying islet cells (insulin-labelled β cells) to then refine it for further downstream analyses to assess the subcellular distribution of key β cell proteins.

Firstly, we determined the most suitable approach for use in cell segmentation of human pancreatic islet images. We evaluated the performance of two deep learning methodologies, the Fully Convolutional Network (FCN) and Residual Neural Network (RNN). Testing involved the use of publicly available cell image data (670 labelled training images, and 65 test images of segmented nuclei) [44], pancreatic islet data (855 training images and 606 test images) [36] and a transfer learning approach involving pre-training using the public cell image data then pancreatic islet images.

**Figure 2:**
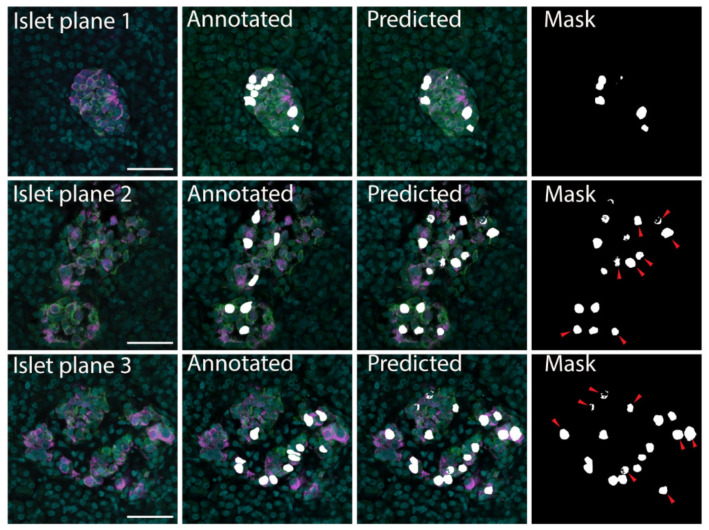
β cell prediction using U-Net deep learning approaches. Examples of microscopy images (Islet plane), the related manually segmented mask overlayed on the original image (Annotated), and the predicted mask image overlayed on the original image (Predicted). The predicted mask (Mask) shows predicted β cells that were not labelled in the annotated image with red arrows. Insulin—magenta, Syntaxin 1A—green, DAPI—cyan, β cell masks—white. Scale bar represents 50 µm.

The datasets were divided into training and validation datasets by *K* partitions where the model is trained on *K-1* and evaluated on the remaining data [45]. The models were tested using 10-fold cross validation with the results listed in Table 1. The FCN-based U-Net model trained using pancreatic islet images only was determined to be the most effective, with accuracy 0.9773, loss 0.0586 and precision 0.5920 (Table 1); therefore, this model was implemented for downstream analyses. The U-Net model was able to correctly identify cells in the original training images. In addition, with new images the U-Net model confirmed the cells that had been manually identified as beta cells (Figure 2). The U-Net model also identified additional insulin-labelled β cells present within the images (Figure 2), suggesting the ability of the model to learn and then predict cells.

### 2.3. Using Machine Learning to Model β Cells within Islets in 3D

**Figure 3:**
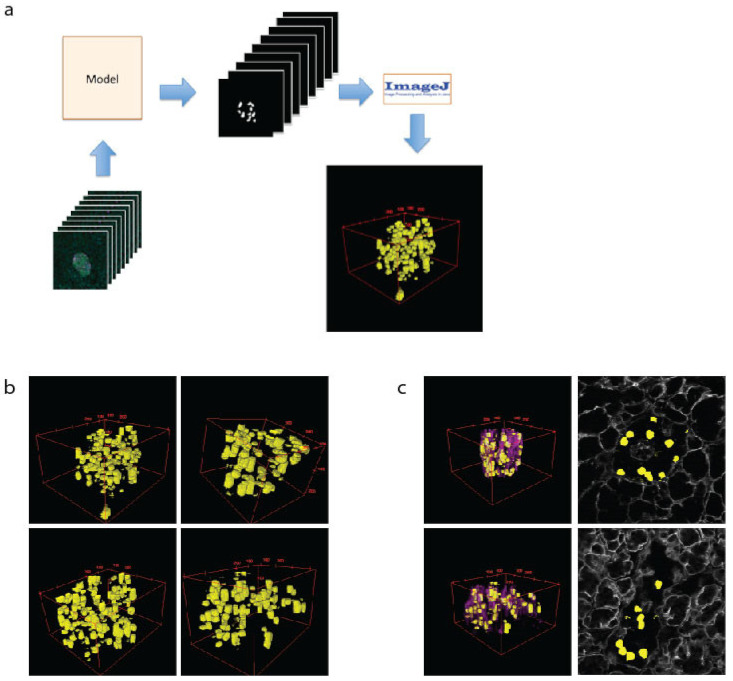
The 3D modelling of β cells within pancreatic islets. (**a**) Schematic of workflow used to create 3D representation of islets using cell masks. (**b**) Examples of 3D models of β cells (yellow) within islets. (**c**) The 3D models showing β cells and the vasculature (as labelled with laminin-magenta) and a single plane showing β cell location (yellow) and vasculature (grey).

The 3D modelling of islets is important to assess cell–cell and cell–vasculature relationships, which have recently been demonstrated as important to islet function [36]. To create 3D models of islets, the β cells are predicted using the U-Net machine learning approach for every z-plane that was imaged. These files were then loaded into ImageJ as a sequence, scaled and projected using the 3D Viewer plugin (Figure 3a). This allows a comprehensive view of cell size and shape (Figure 3b). To assess the spatial relationship of the β cells to the islet vasculature, the laminin images were added to the 2D images or 3D reconstructions (3c, purple). Whilst 3D computational modelling of islets has been performed previously [32,43], our data validate the modelling capacity of our approaches, which we then used in downstream applications.

### 2.4. Using Machine Learning to Assess Subcellular Proteins within β Cells

**Figure 4:**
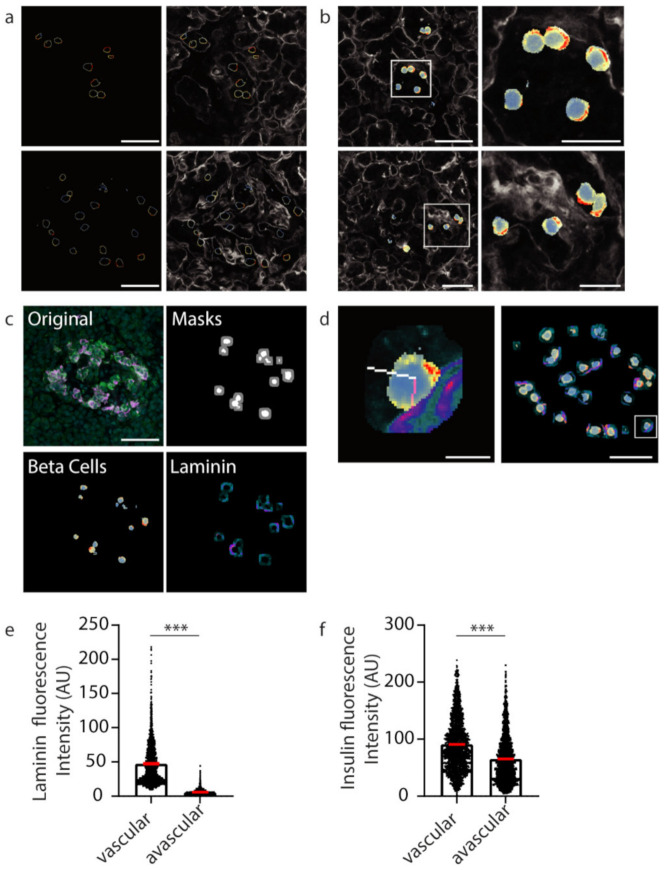
Insulin intensity analyses using computational techniques. (**a**) Representative β cell boundaries with insulin fluoresce represented using a defined heatmap LUT in ImageJ with vasculature (laminin-grey). Heat map fluorescence low to high; blue to red. (**b**) Heat maps applied to whole β cells within islets (**c**) Original islet image with masks for cells (white) and cell boundaries (grey), heatmap for insulin (Beta cells; fluorescence low to high; blue to red) and laminin (Laminin; fluorescence low to high; teal to orange). (**d**) A 10 pixel-wide scan line from the vasculature (laminin high intensity) in pink was used to determine insulin florescence intensity. In white is the line used to measure insulin intensity from the avascular cell boundary (low laminin intensity). The location of the example cell in the whole islet image is shown using a white box. (**e**) Graph of laminin intensity at the vascular and avascular regions. (**f**) Graph of insulin intensity at the vascular and avascular regions. *** *p* < 0.001. Scale bar represents 50 µm for whole islets, 20 µm for insets in (**b**) and 5 µm for the inset in (**d**).

After demonstrating that our U-Net-based deep learning approach can successfully identify insulin-positive β cells within islets from image files, we then wanted to investigate the subcellular staining profiles of proteins of interest within β cells. Firstly, to identify individual β cells, instance segmentation was performed on the semantic segmentation of the predicted β cells. Once individual cell boundaries were identified, the fluorescence values were extracted and presented as a heat map (blue to red; low to high) (Figure 4a). An overlay of the β cell boundary heat map image onto the laminin channel image can be used to assess whether proteins are polarised towards the islet cell vasculature.

Extracting the fluorescent values of insulin staining from the whole cell mask gives insight into the subcellular localisation and concentration of insulin with respect to the vasculature (Figure 4b). To develop an unbiased, automated approach involving a line-scan originating from regions of high and low laminin concentration near the β cell boundary representing the vascular and avascular faces, respectively, software was developed to automatically identify a 10-pixel boundary region (Figure 4c, grey) around the predicted β cells (Figure 4c, white), and locate points of high and low laminin concentration in this boundary region by scanning using a 9 × 9 pixel window (Figure 8) representing the cell circumference. The software then determined the pixel locations of a line-scan from these high and low laminin points towards the cell centre (Figure 4d, vascular region: pink line; avascular region: white line).

The mean fluorescence intensities of insulin staining along the line-scans were determined and this produced statistical data for 2365 predicted β cell instances (cells within individual planes). The vascular face was identified with significantly higher laminin signal than the avascular face (Figure 4e, *** *p* < 0.001; vascular laminin 47.41 ± 30.89 avascular laminin 5.807 ± 4.264). At this computationally identified vascular face a significantly higher concentration of insulin was observed than at the avascular face (Figure 4f, *** *p* < 0.001; vascular insulin 90.78 ± 46.57, avascular insulin 65.22 ± 42.3). These data recapitulate the results from the manually analysed data in Figure 1. However, now the deep learning approach was able to generate statistical data for over 2000 β cell instances to investigate the polarisation of β cells in an unbiased and timely manner.

## 3. Discussion

We found that machine learning approaches are useful in the analysis of large datasets and can be applied to facilitate an understanding the organisation of sub-cellular structures. In this example, we show that the conclusion reached by machine learning algorithms is coincident with that from manual analysis, and both methods show that insulin contents within individual β cells are enriched at the β cell–vascular interface.

The importance of the automated approach is that it is unbiased and drawn from a much bigger dataset than is reasonably feasible with a manual approach. This not only is useful in terms of time efficiency and the increase in the number of example cells that are analysed, but it also demonstrates that an automated approach, where only a few initial quantitative constraints are placed on the model, can confirm the results from a manual approach, which is driven by user expertise. This is important for complex structures, such as islets of Langerhans, where there can be ambiguous images where we currently rely on experts for interpretation. If, through machine learning, we do not need such expert input, then this further underscores the robustness and reproducibility of the findings. It is interesting that the U-Net approach identified β cells that were not found in the manual approach, which suggests either inaccuracies in the algorithm or in the expert identification of the cells. In this context, it is important to note that in any single image plane β cells will be sectioned randomly. Those cells sectioned through the middle will show the clearest, most obvious insulin staining, whereas cells sectioned at their periphery could show fragmented insulin staining, making their identification problematic. Thus, while we expect that the U-Net learning approach might misidentify some β cells (as would an expert), there are still advantages of being able to sample across large volumes and include large numbers of cell instances.

The U-Net modelling approach achieved very high precision with the public learning image data. The images used in the training were DAPI-stained nuclei that have a very consistent morphology both within a single image and across datasets. In contrast, insulin-stained β cells within islets of Langerhans have quite a different morphology, reflecting the close-packing of the cells around the complex network of capillaries. We believe that the diversity in β cell morphology is the basis of the reduced precision in the images of the islets.

The image segmentation applied in our approach is applicable to the identification of the organisation of any subcellular compartment. In the example used here, β cells orientate consistently with respect to capillaries, and therefore identification of the capillary contact provides a spatial point of reference around which the distribution of other compartments or proteins can be mapped. However, it is common for most tissues to show a characteristic organisation, and therefore, with an appropriate external point of reference, such as a lumen or contact with basement membrane, exactly the same approach we use here will be applicable.

We conclude that machine learning is a valuable approach to the analysis of sub-cellular structures within the complex architecture of an organ. In the example here, the approach has enabled a far larger dataset than is practical through manual segmentation, and the results add further evidence for the polarisation of β cells.

## 4. Materials and Methods

### 4.1. Human Pancreas Samples

Human pancreatic samples were processed via methods previously described [36]. In brief, tissue was sourced from pancreatic tumour resections (with patient consent, approved by the Northern Sydney Local Heath District Human Research Ethics Committee) or cadaveric donors (study approved by the Human Research Ethics Committee at the University of Sydney). Tissue samples were fixed in 4% paraformaldehyde then mounted in 1.5% low-melting point agarose and 150 µm sections were cut on a vibratome as described by Marciniak et al. [41]. Free-floating sections were stained as described by Meneghel-Rozzo et al. [46]; this involved incubations in blocking buffer (3% BSA, 3% donkey serum, 0.3% Triton X-100) for 4 h at room temperature, and then in primary antibody at 4 °C for 16 h. Sections were washed in 1X PBS and secondary antibody with DAPI incubations were for 5 h at room temperature. After washing, the sections were mounted using ProLong Diamond Antifade (Thermo Fisher Scientific) and imaged on a Leica SP8 confocal microscope using the 63X objective (Leica Microsystems, Wetzlar, Germany).

### 4.2. Quantification of Insulin Intensity

Image analysis was performed using Fiji (ImageJ) [47] and Metamorph 7.8 (Molecular Devices, San Jose, CA, USA). Graphs were produced using GraphPad Prism v7.02 (GraphPad Software, San Diego, CA, USA). We identified β cells (insulin staining) making contact with the vasculature (laminin staining) and β cell boundaries were identified using Syntaxin 1A staining. To analyse insulin intensity, a line-scan was drawn from the vascular face to the avascular face of the cell and the average intensity across the line extending from the Syntaxin-labelled cell boundary for 2 µm into the cell was measured from each face.

### 4.3. Statistical Analyses

Statistical analyses were performed using GraphPad Prism. A paired two-tailed student’s t test was used to analyse insulin intensity (Figure 1). A paired two-tailed student’s t test was used to analyse insulin and laminin intensity (Figure 4). Data are expressed in the text as mean ± SEM.

### 4.4. Imaging Datasets

Human pancreatic islet images: The dataset consisted of confocal microscopy images stained with insulin, syntaxin 1A, laminin and DAPI [36]. The data were produced with the Lecia SP8 confocal microscope using the 63*X* objective. Each islet consisted of between 50 and 90 z-stacked images. The images were 2048 × 2048 pixels in size, and the dimensions of each voxel (*x*, *y*, *z*) were 0.0901 × 0.0901 × 0.3362 micron^3^. The data are 8-bit grey scale. The training images were selected from each of the series spaced five slices apart, sufficient to encompass large variations in the images. These data consisted of 855 training images and 606 test images.

Segmented nuclei images: Image set BBBC038v1 from the Broad Bioimage Benchmark Collection Cacicedo et al. [44]. This dataset consisted of 675 training images and 65 test images.

### 4.5. Image Format Conversion

The human pancreatic islet images were originally stored in Leica image file (LIF) format (.lif files). The LIF files were opened in ImageJ/Fiji and the required channels were combined into a composite image that was saved as a PNG file. An ImageJ/Fiji macro was developed to quickly generate PNG files for all image slices to be used in the deep learning model (Supplementary Information S1). Images were resized to 512 × 512 pixels as this size was determined to be optimal to maintain accuracy, while fitting within the limitations of computing resources available.

### 4.6. Training Data-Manual Annotation

**Figure 5:**
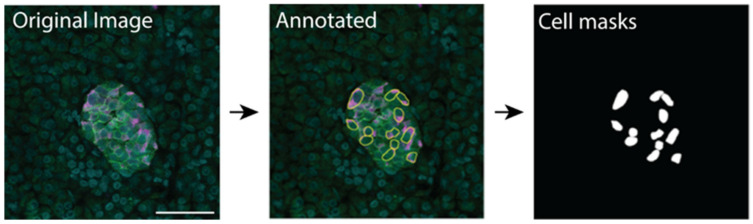
Manual annotation of images using ImageJ and resulting masks used for training the machine learning model. Scale bar represents 50 µm.

Training data were manually labelled to produce mask files for use in training and for evaluating a supervised learning deep learning model. The cell boundary was manually traced in ImageJ/Fiji using insulin staining to identify β cells, syntaxin staining to identify the cell boundary and DAPI staining to identify the cell nucleus; the annotation was then used to create a cell mask (Figure 5). A basic ImageJ macro to improve efficiency was developed (Supplementary Information S2). The image annotation was performed by a student working under the supervision of PhD-level cell biologists.

### 4.7. Training Data-Image Augmentation

**Figure 6:**
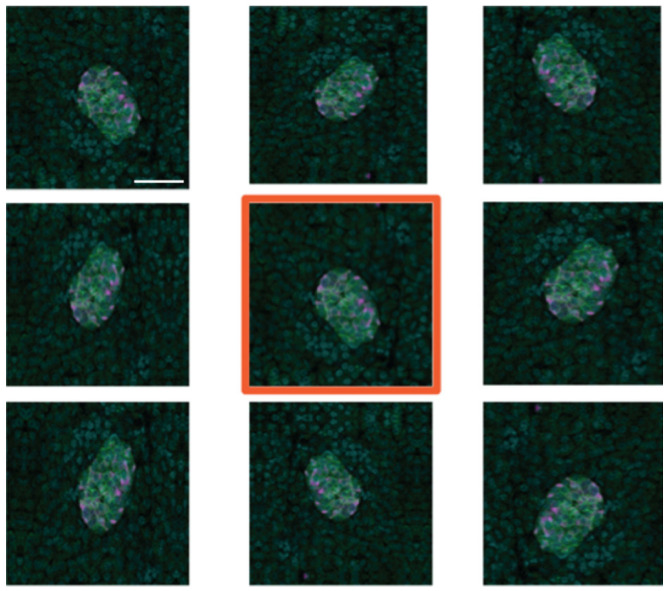
Example of image augmentation used to increase image numbers in the training dataset. The original image has an orange border. Scale bar represents 50 µm.

To create additional training data, we used image augmentation using the Python Keras image data generator. Each of the 95 manually created training images was subject to eight iterations of random transformations, including horizontal and vertical mirroring (or flipping), shearing and shifting (horizontally and vertically), and rotation (Figure 6). This resulted in the creation of 760 additional images, which, along with the original 95, is sufficient for training the deep learning models developed.

### 4.8. Model Development and Testing

An FCN model, based on U-Net [48], was built using the Python Keras high-level neural network API library, based on an example that used publicly available cell image data consisting of 670 labelled segmented nuclei images [19]. The ResNet RNN model was built in Python using the Keras library and was based on example implementations [49,50]. ResNet was computationally expensive with a scaling up of CPU and required memory in comparison to U-Net.

### 4.9. 3D Models of β Cells within Islets

The best performing FCN model that had been trained on 855 training images was then applied to the test images to make predictions of β cell locations (semantic segmentation). The predicted β cell mask images generated for each series were then loaded into ImageJ/Fiji as a single image sequence. The 3D representations were created using the 3D Viewer plugin in ImageJ/Fiji. The image stack was scaled to match the original voxel ratios (1:1:3.7) to create a realistic depiction of the islet in three dimensions. The voxel dimension from the imaging files was 0.0902 × 0.0902 × 0.3362 microns (*x*, *y*, *z*).

### 4.10. Instance Segmentation of β Cells

**Figure 7:**
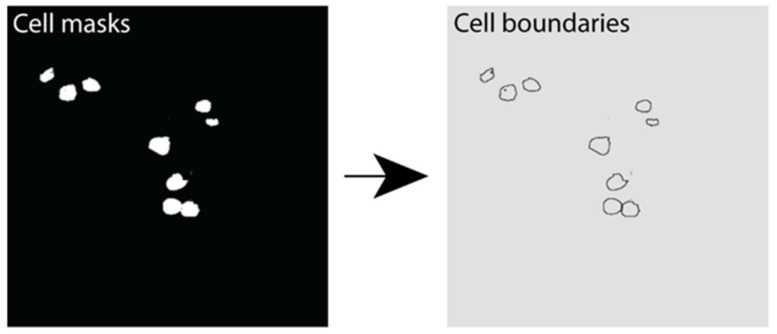
Instance segmentation output showing β cell boundaries. Example image outputs showing cell masks (white) and the resulting cell boundaries predicted using instance segmentation in Python.

Instance segmentation was performed using the marker-controlled watershed transform [51,52]. This was performed using the Python library Scikit-Image library. A Gaussian filter was applied to reduce noise in cell detection. The resulting output was an image with each cell boundary assigned a label representing its unique cell instance number (Figure 7).

### 4.11. Identifying the Vascular and Avascular Regions and Assessing β Cell Subcellular Insulin Fluorescence Values

After predicting cell location and boundary via instance segmentation, we then created a boundary region mask to extract protein intensity values from the appropriate channel in the microscopy images. Python image processing library routines (“scikit-image”) were used to perform a binary dilation of each predicted β cell mask, and then subtraction of the original mask.

**Figure 8:**
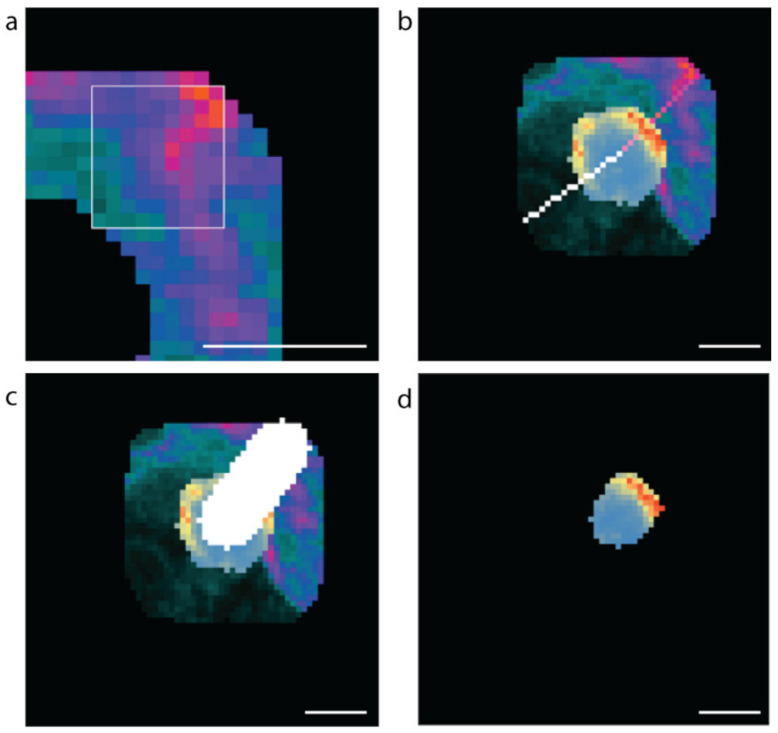
Methods used to computationally assess vascular and avascular regions. (**a**) The 9 × 9-pixel window used for determining the high and low mean laminin points in the cell boundary region, (**b**) the software generated scan lines from the high (pink) and low (white) laminin concentration points to the cell centre. (**c**) The 10-pixel-wide scan-line and (**d**) the insulin concentrations along the 10-pixel-wide scan line within the β cell (blue low to red high insulin staining intensity). Scale bar represents 5 µm.

A 9 × 9-pixel window was scanned horizontally and then vertically across the boundary region to identify the vascular (high laminin) and avascular (low laminin) regions (Figure 8a). The high or low concentration point is taken to be the centre of the 9 by 9 grid. Once the regions of interest are determined, lines to the cell centre are drawn (Figure 8b). For each coordinate on each scan-line within the β cell, the mean insulin value is calculated for a 10-pixel by 10-pixel region around that point, and the maximum value is recorded for analysis (Figure 8c,d). Only insulin values for pixels that were within the predicted β cell were used in determining the maximum mean insulin concentration. The resulting fluorescence values were exported into csv files. The data were filtered to exclude β cells instances with a radius of less than 5 µm; therefore, all cell instances greater than 490 pixels were used in the analyses.

## Figures and Tables

**Table 1:** Testing results from deep learning methodologies ^1^.

Model	Data	Accuracy	Loss	Precision	Recall	F1	Epoch
U-Net	Public	0.9750	0.0628	0.9207	0.9072	0.9125	54.6
	Islet	0.9773	**0.0586**	**0.5920**	0.1308	0.2012	19.5
	Transfer/ Islet	**0.9777**	0.0594	0.5828	0.1407	0.2189	22.3
ResNet	Public	0.9640	0.0933	0.9022	0.8664	0.8821	37.0
	Islet	0.9764	0.0624	0.5267	0.2081	0.2852	19.0
	Transfer/ Islet	**0.9765**	**0.0622**	**0.5288**	0.1688	0.2442	15.8

^1^ The best values achieved for each model are displayed in bold text. Table descriptors defined in Supplementary Table S1.

## Data Availability

The data presented in this study are available in the supplementary material.

## Supplementary Materials

The following are available online at https://www.mdpi.com/article/10.3390/metabo11060363/s1, Table S1: Definitions of the descriptors used to explain the performance of deep learning models, Information S1: Image Processing Macros: Image format conversion, Information S2: Image Processing Macros: Processing Cell Masks.
