# Supplementary material for: Machine Learning Algorithms, Applied to Intact Islets of Langerhans, Demonstrate Significantly Enhanced Insulin Staining at the Capillary Interface of Human Pancreatic β Cells
Source: Metabolites. 2021 Jun 7;11(6):363. doi: 10.3390/metabo11060363 (PMC8229564; doi:10.3390/metabo11060363)
Supplement: Supplementary file 1 [file metabolites-11-00363-s001.zip › metabolites-1227201-supplementary.pdf]

Supplementary Material

Supplementary Table S1: Definitions of the descriptors used to explain the performance of deep learning models

Descriptor	Definition
Accuracy	Ability to predict true positives and true negatives in the data
Loss	Loss is a measurement of the gap between what values have been predicted by the model and what is the true value as provided by the training data.
Precision	Indication of models’ ability to predict the location of true positives as a proportion of true positives and false positives
Recall	Indication of the models’ ability to predict the location of true positives as a proportion of true positives and false negatives
F1	An overall metric combining precision and recall
Epoch	Average number of learning iterations undergone by the before no further improvement was achieved

S1- Image Processing Macros: Image format conversion

```
macro "Create Slice Images" {

// Display welcome dialogue box
	Dialog.create("Convert a Project Series into individual slice images");

	Dialog.addMessage("This macro allows you to:\n 1) Import a .lif file\n 2) Create individual images for each slice");

	Dialog.addRadioButtonGroup("Continue?", newArray("Yes", "No"), 1,2,"Yes");

	Dialog.show();

	Continue = Dialog.getRadioButton();

	if(Continue == "No") {

		exit();

	}


// Select and open the .lif imagefile

	open();


//Adjust the channel colours


	run("Channels Tool...");
```

```
	Stack.setDisplayMode("color");

	Stack.setChannel(1);

	run("Magenta");

	Stack.setChannel(2);

	run("Blue");

	Stack.setChannel(3);

	run("Green");

	Stack.setChannel(4);

	run("Cyan");

	Stack.setChannel(5);

	run("Grays");

// Create a composite image and select only channels 1,3,5

	Stack.setDisplayMode("composite");

	Stack.setActiveChannels("10110");

// Change type to RGB

	run("RGB Color", "slices keep");

// Display close dialogue box

	Dialog.create("All Done");

	Dialog.addMessage("Image is ready to be saved using:\n     File>Save As>Image Sequence...\n Format=PNG; Digits=3;\n Name: replace from .lif with -SeriesNN-")

	Dialog.show();
```

S2- Image Processing Macros: Processing Cell masks

```
macro "Save the masks and ROIs for cell boundary segmentation [L]" {

/

// Set variables

        title = getTitle()

// Clear selections from the image: “Image>Overlay>Remove Overlay”

        run("Remove Overlay");

// Create a black and white mask image of the selected cell

        run("Create Mask");

// Saves the mask to a .png file: “File>Save as>PNG…”

        saveAs("PNG");
        close();


 // Saves the selection in the ROI Manager: “Image>Overlay>Add Selection>”

        selectWindow(title);
        run("Add Selection...");

// Load the previous selections: “Image>Overlay>From ROI Manager”

        run("From ROI Manager");


// Save all the selections:  “Image>Overlay>From ROI Manager”

        run("To ROI Manager");


//
        close();
        exit();
}
```
